# Food protein-derived amyloids do not accelerate amyloid β aggregation

**DOI:** 10.1038/s41598-023-28147-5

**Published:** 2023-01-31

**Authors:** M. Mahafuzur Rahman, Rodrigo Sanches Pires, Anja Herneke, Vasantha Gowda, Maud Langton, Henrik Biverstål, Christofer Lendel

**Affiliations:** 1 Department of Chemistry, KTH Royal Institute of Technology, Teknikringen 30, 100 44 Stockholm, Sweden; 2 Department of Molecular Sciences, Swedish University of Agricultural Sciences, BioCentrum, Almas Allé 5, 756 61 Uppsala, Sweden; 3 Department of Biosciences and Nutrition, Karolinska Institutet, NEO/Floor 8, Blickgången 16, 141 52 Huddinge, Sweden

**Keywords:** Prions, Protein aggregation, Kinetics

## Abstract

The deposition of proteins in the form of amyloid fibrils is closely associated with several serious diseases. The events that trigger the conversion from soluble functional proteins into insoluble amyloid are not fully understood. Many proteins that are not associated with disease can form amyloid with similar structural characteristics as the disease-associated fibrils, which highlights the potential risk of cross-seeding of disease amyloid by amyloid-like structures encountered in our surrounding. Of particular interest are common food proteins that can be transformed into amyloid under conditions similar to cooking. We here investigate cross-seeding of amyloid-β (Aβ), a peptide known to form amyloid during the development of Alzheimer’s disease, by 16 types of amyloid fibrils derived from food proteins or peptides. Kinetic studies using thioflavin T fluorescence as output show that none of the investigated protein fibrils accelerates the aggregation of Aβ. In at least two cases (hen egg lysozyme and oat protein isolate) we observe retardation of the aggregation, which appears to originate from interactions between the food protein seeds and Aβ in aggregated form. The results support the view that food-derived amyloid is not a risk factor for development of Aβ pathology and Alzheimer’s disease.

## Introduction

The structural characteristics of proteins are intimately connected with their functional and dysfunctional properties. Although this statement most often refers to the native, globular state of the protein molecules, it is as valid also for alternative states, such as the amyloid state. This state, which is signified by ordered, β-sheet rich filamentous aggregates of proteins, was historically primarily associated with disorders such as Alzheimer’s disease, Parkinson’s disease, type 2 diabetes as well as prion diseases^1–4^. Today it is established as a generic state that any protein chain could adopt, given the right circumstances^5^, and there is a growing number of examples of amyloid structures that provide essential biological functions to various organisms, from bacteria and yeast to plants and mammals^6^. Amyloid-like protein nanofibrils have also found their way into engineering and materials science as a versatile nanoscale building block for functional and sustainable materials^7,8^. Moreover, the formation of protein-based gels is often associated with the assembly into amyloid-like fibrils^9,10^. This property together with their ability to serve as thickener or stabilizers of foam or emulsions has highlighted protein nanofibrils as highly interesting ingredients for the food industry^11,12^. The nanofibrils are also investigated with the purpose of design of novel protein-rich foodstuff, e.g. to replace meat products^13^.

The transition from being closely associated with disease to the new role as promising building blocks in sustainable materials and food raises the question about the potential health risks of protein nanofibrils. There are so far only a few studies that have investigated the direct toxicity of non-disease amyloid-like nanofibrils in cell culture^14–16^ and in rats^17^ and no acute toxic effects were observed. However, the fibrils could also have more long terms effects. The most well-known example is transmissible spongiform encephalopathies caused by the prion proteins, which can be transmitted through ingestion of prion-containing substances (both within and across species borders). There are indeed reports of amyloidosis in animal models triggered by oral intake of amyloid-containing materials^18–20^.

The aggregation of amyloid β (Aβ), and in particular the 42 amino acid residues long variant Aβ_1–42_, into senile plaques is a central pathological signature of Alzheimer’s disease^21^. A number of in vitro studies have explored the cross-talk between Aβ and other human disease-associated amyloid proteins and acceleration of Aβ aggregation has been reported for α-synuclein^22,23^, AA amyloid^24^ and IAPP^25^. Some studies also go beyond the disease-associated proteins and investigate cross-seeding of Aβ by functional amyloid^26,27^ or non-amyloid protein fibers^25^. The majority of these studies find that Aβ in vitro aggregation is accelerated by the presence of other types of amyloid. Hence, there may be a connection between exposure to endogenous amyloid and the onset of Aβ pathology.

We have in recent studies demonstrated the ability of several food proteins, as well as peptides derived from these proteins, to form amyloid-like fibrils^13,28–32^. Here we use this knowledge to investigate the abilities of a range of food protein-derived amyloid fibrils to accelerate the aggregation of Aβ in carefully executed kinetics experiments. Interestingly, we find that none of the investigated amyloids were able to accelerate fibril formation of Aβ_1–42_, which support the assumption that it is safe to use amyloid-like protein nanofibrils in food and materials.

## Results and discussion

### Aβ fibrillation kinetics

**Figure 1:**
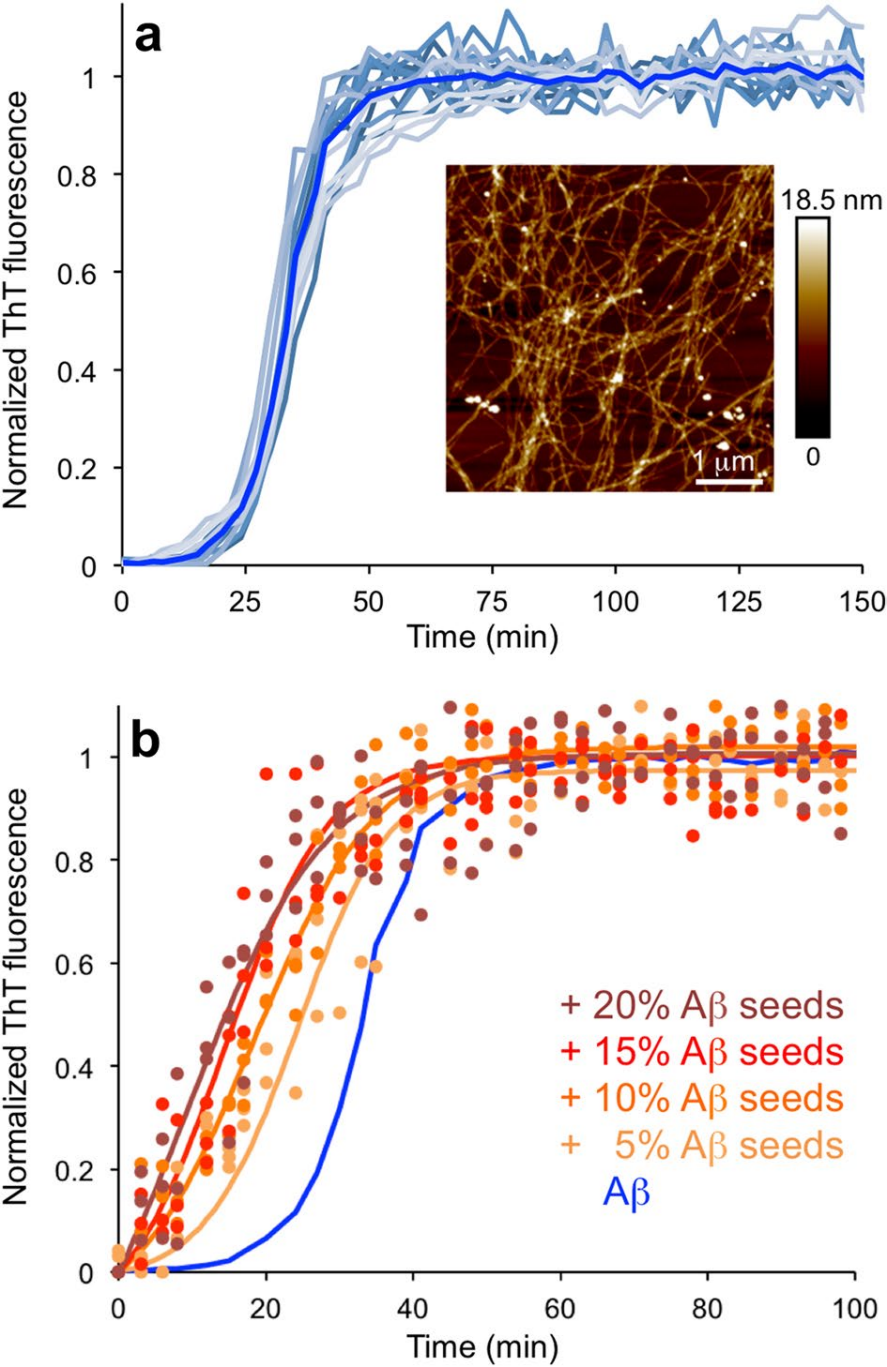
Aggregation of Aβ_1–42_ with and without Aβ_1–42_ seeds. (**a**) ThT kinetics of Aβ_1–42_ without any added seeds. The figure shows 15 traces from 5 experiments and the average in clear blue. Inset: representative AFM image of Aβ_1–42_ amyloid fibrils. (**b**) ThT kinetics of Aβ_1–42_ with 5%, 10%, 15% or 20% Aβ_1–42_ seeds added. The average data for Aβ_1–42_ without seeds is shown in blue. Experimental data for three replicates at each condition (filled circles) and sigmoidal curve fits of the average data (solid lines) are shown.

Recombinant Aβ_1–42_ was produced as a fusion with NT solubility tag as described previously^33^. The purified peptide from this production protocol has been thoroughly characterized in previous work. For the peptide batch used in this work, the assembly of the peptide into amyloid fibrils was confirmed by increased ThT fluorescence and the observation of fibrillar structures by AFM (Fig. 1a). Fibrillation kinetics experiments were performed at 37 °C with 3 μM Aβ_1–42_ in HEPES buffer pH 8, 140 mM NaCl and 15 μM ThT. The reproducibility of these kinetics experiments was high as illustrated in Fig. 1a showing in total 15 traces from 5 different experiments. The *t*_*1/2*_ is 33.5 ± 1.9 min.

Previous studies of Aβ_1–42_ fibrillation kinetics have shown that the mechanism is dominated by secondary nucleation^34^. We used the AmyloFit modeling framework^35^ to verify that our data was consistent with such model and found that it could be fitted to a secondary nucleation dominated model with rate constants similar to those reported in previous literature (data not shown). Hence, the kinetics results presented here should be comparable with other studies in the field.

Preformed Aβ_1–42_ amyloid fibrils were used as a positive control for the fibrillation kinetics experiments. The addition of 5–20% of Aβ_1–42_ seeds (i.e. sonicated fibrils) resulted in increased fibrillation rates (Fig. 1b) with *t*_*1/2*_ between 24.7 ± 2.1 min (5%) and 11.8 ± 3.7 min (20%). These results show that the employed experimental protocol exhibits faster assembly kinetics for seeded reactions, as expected.

### Seeding by fibrils prepared from food proteins

**Figure 2:**
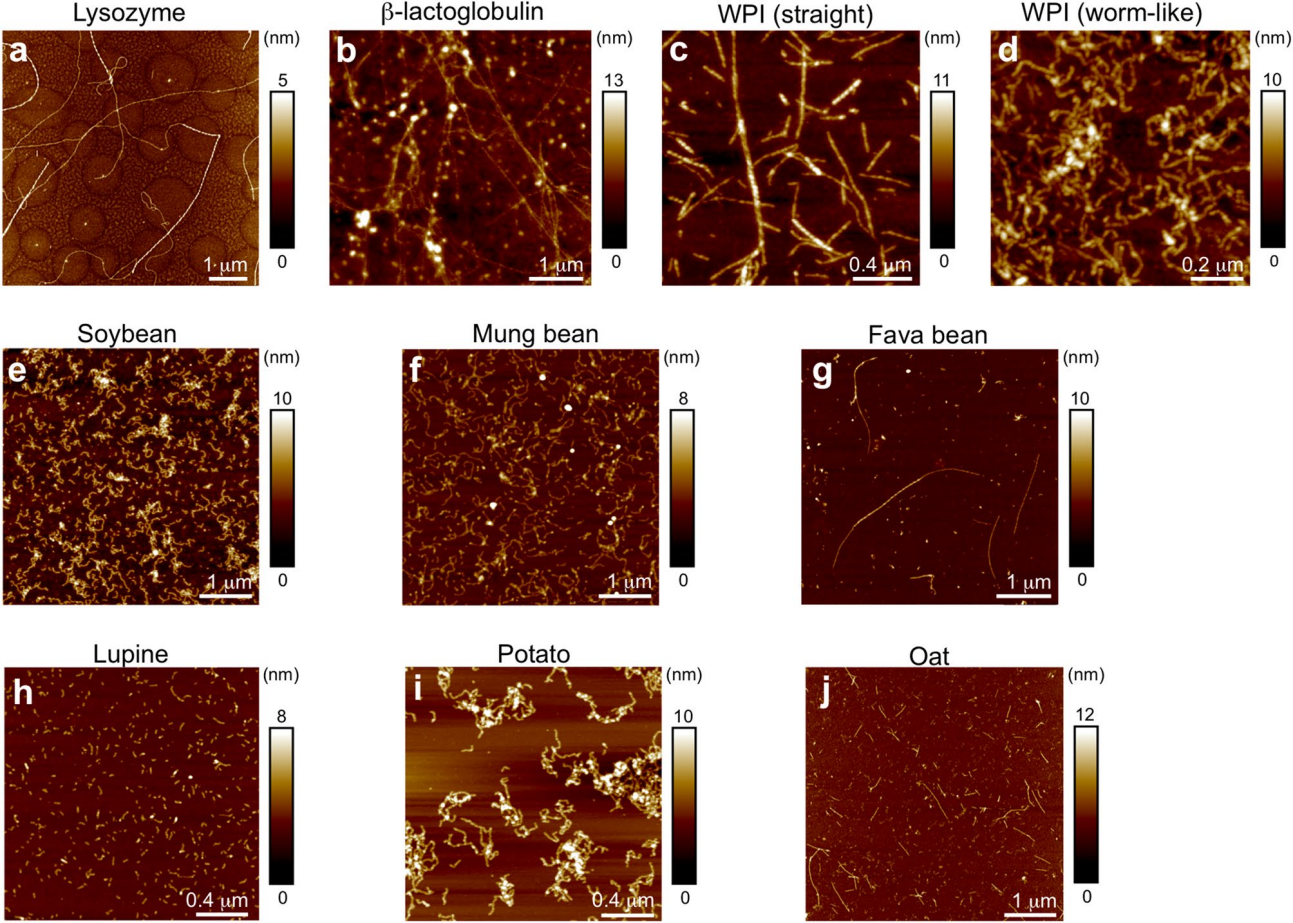
AFM images of amyloid fibrils from the different protein sources used to prepare seeds for the study. (**a**) Lysozyme, (**b**) pure β-lactoglobulin, (**c**) WPI (straight morphology), (**d**) WPI (worm-like morphology), (**e**) soybean, (**f**) mung bean, (**g**) fava bean, (**h**) lupine, (**i**) potato, and (**j**) oat.

Amyloid-like fibrils were prepared from nine different food protein sources, including hen egg (lysozyme), bovine milk (pure β-lactoglobulin and whey protein isolate, WPI), four variants of legumes (soybean, mung bean, fava bean, and lupine), potato protein isolate and oat flour. The fibrillation protocols for all the resources have been reported and the resulting fibrils characterized in previous studies^13,29–32^. This information is summarized in Table 1. Representative AFM images of fibril preparations from the different protein resources are shown in Fig. 2. Amyloid seeds were prepared by sonication of the fibril dispersions and ThT kinetics were recorded at two seed concentrations, corresponding to 5% and 10% of the Aβ_1–42_ concentration (by mass).

**Table 1:** Protein resources used for fibril seeds.

Protein	Source	Dominating fibril morphology	Ref. fibril formation/characterization
Lysozyme	Sigma	Long, straight or bended	
β-lactoglobulin	Sigma	Long, straight	
β-lactoglobulin	WPI	Long, straight	^32,36^
β-lactoglobulin	WPI	Short, curved (worm-like)	^32,36^
Soybean	SPI	Short, curved	^30^
Mung bean	Whole beans	Long, curved	^13^
Fava bean	Whole beans	Long, straight	^13^
Lupine	Seeds	Short rods	^13^
Potato	PPI	Short, curved	^31^
Oat	Flour	Medium length, straight	^13^

**Figure 3:**
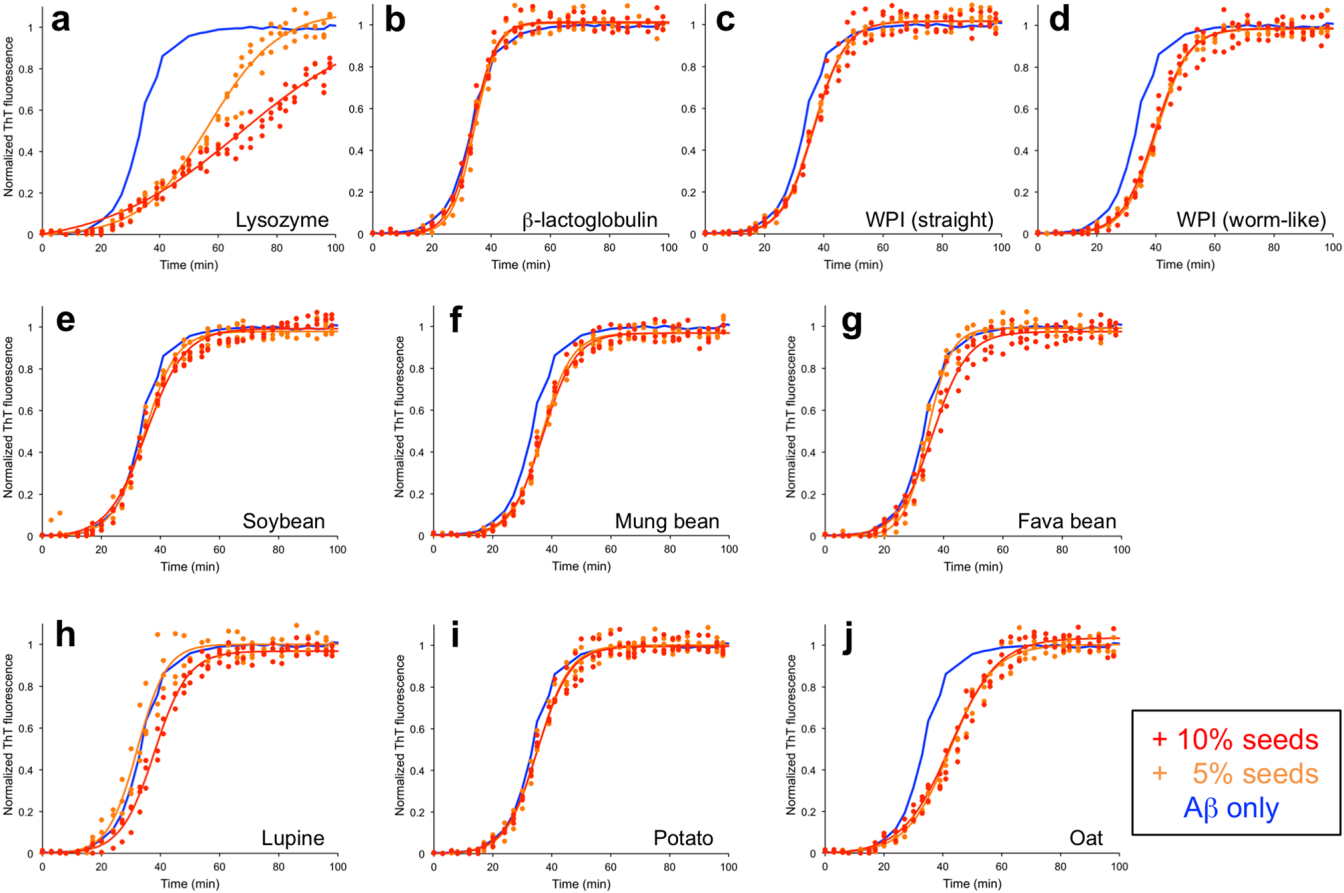
ThT kinetic traces for Aβ_1–42_ without seeds (blue) and seeded with 5% (orange) or 10% (red) seeds from (**a**) lysozyme, (**b**) pure β-lactoglobulin, (**c**) WPI (straight morphology), (**d**) WPI (worm-like morphology), (**e**) soybean, (**f**) mung bean, (**g**) fava bean, (**h**) lupine, (**i**) potato, and (**j**) oat. Experimental data for three replicates at each condition (filled circles) and sigmoidal curve fits of the average data (solid lines) are shown.

Hen egg *lysozyme* constitutes *ca* 3.5% of the proteins in hen eggwhite^37^. It is a frequently used model system for amyloid formation, which also has a connection to human disease as mutations in human lysozyme can lead to amyloidosis^38^. The morphology of the amyloid prepared from lysozyme includes long straight fibrils but also some with a higher degree of curvature (Fig. 2a). Interestingly, the ThT kinetics (Fig. 3a) indicates that lysozyme seeds may have an inhibiting effect on Aβ_1–42_ fibrillation. This is in contrast to the reported acceleration of α-synuclein fibrillation^39,40^. The explanation for this difference may be related to the high isoelectric point (pI > 11) of lysozyme and lysozyme amyloid fibrils^40,41^ that means that they will be positively charged at pH 8. In the case of α-synuclein that leads to release of the protecting intramolecular interactions from the negatively charged C-terminus and thereby increased aggregation rate^42,43^. For Aβ, it can be speculated that the positively charged lysozyme seeds may bind to the negatively charged Aβ (as monomer or in aggregated state) and thereby counteract the assembly process.

Another frequently used model system for amyloid formation, as well as an important protein in many food applications, is *β-lactoglobulin.* This is the main component of the whey fraction from milk, which is a frequently utilized food ingredient. We explored the effect of amyloid seeds from commercial, pure β-lactoglobulin as well as fibrils prepared from WPI (which are formed by the same protein^44^). It has previously been shown that β-lactoglobulin can form fibrils of distinct morphologies depending on the initial protein concentrations of the samples^32,45^. At lower concentrations, long, straight fibrils are formed while higher concentrations result in short, curved (worm-like) fibrils. Both morphologies are indeed amyloid-like as they display typical biophysical characteristics of such fibrils, including a cross-β pattern in X-ray fiber diffraction experiments^32,46^. Our investigation included both types of fibril seeds prepared from WPI. The results show that amyloid seeds prepared from pure β-lactoglobulin do not affect the aggregation kinetics of Aβ_1–42_ at all (Fig. 3b). For the WPI-derived fibrils, a weak inhibitory effect is observed (Fig. 3c,d). However, there is no clear concentration dependence of the inhibition and the observed differences are within error range of the measurements.

Among plant protein-derived amyloid, legumes are the most frequently investigated. We included four legume sources that are known to form amyloid-like fibrils: *soybean*, *mung bean*, *fava bean,* and *lupine*. The soybean fibrils were prepared from an industrial protein isolate while mung, fava, and lupine proteins were extracted and purified in-house^13^. The morphologies of the fibrils vary. Soybean protein isolates mainly form short, curved fibrils but with occasional occurrence of long straight fibrils^30^. Mung bean forms fibrils that are long and curved, a morphology that we have not observed for any other protein resource. The fava bean proteins can assemble into straight fibrils, as those used in this study, or worm-like fibrils as previously reported^13^. The lupine proteins mainly form very short, rod-like structures that are difficult to classify as straight or curved. In the ThT kinetics experiments all four legume amyloid seed were found to have no or weakly inhibiting effects on Aβ_1–42_ aggregation (Fig. 3e–h).

Finally we included amyloid fibrils from two non-legume sources. Potato protein (from an industrial isolate^31^) was shown to form short and curved fibrils (Fig. 2h). Although potato is not a major natural source of protein in our food, there is an increasing interest to use the protein isolate in food applications as it is a sidestream from starch manufacturing. The potato protein amyloid seeds had no effect on the Aβ_1–42_ aggregation kinetics (Fig. 3i). Oat is a grain with a relatively high protein content, which is of special interest for products without wheat gluten. The amyloid fibrils used in this study were prepared from in-house extraction of the protein from oat flour and appear as straight fibrils of medium length (Fig. 2h)^13^. The ThT experimental results show that the oat seeds had an inhibitory effect similar to the one observed for lysozyme, which is higher than the other plant- and whey fibrils (Fig. 3j). In the case of lysozyme, we speculated that the effect may be related to the positive charge of the fibrils. Interestingly, oat globulin has a higher isolectric point (*ca.* 7)^47^, than most of the other investigated protein sources. However, it is still lower than the pH used for the aggregation assay (pH 8).

### Inhibition mechanisms

**Figure 4:**
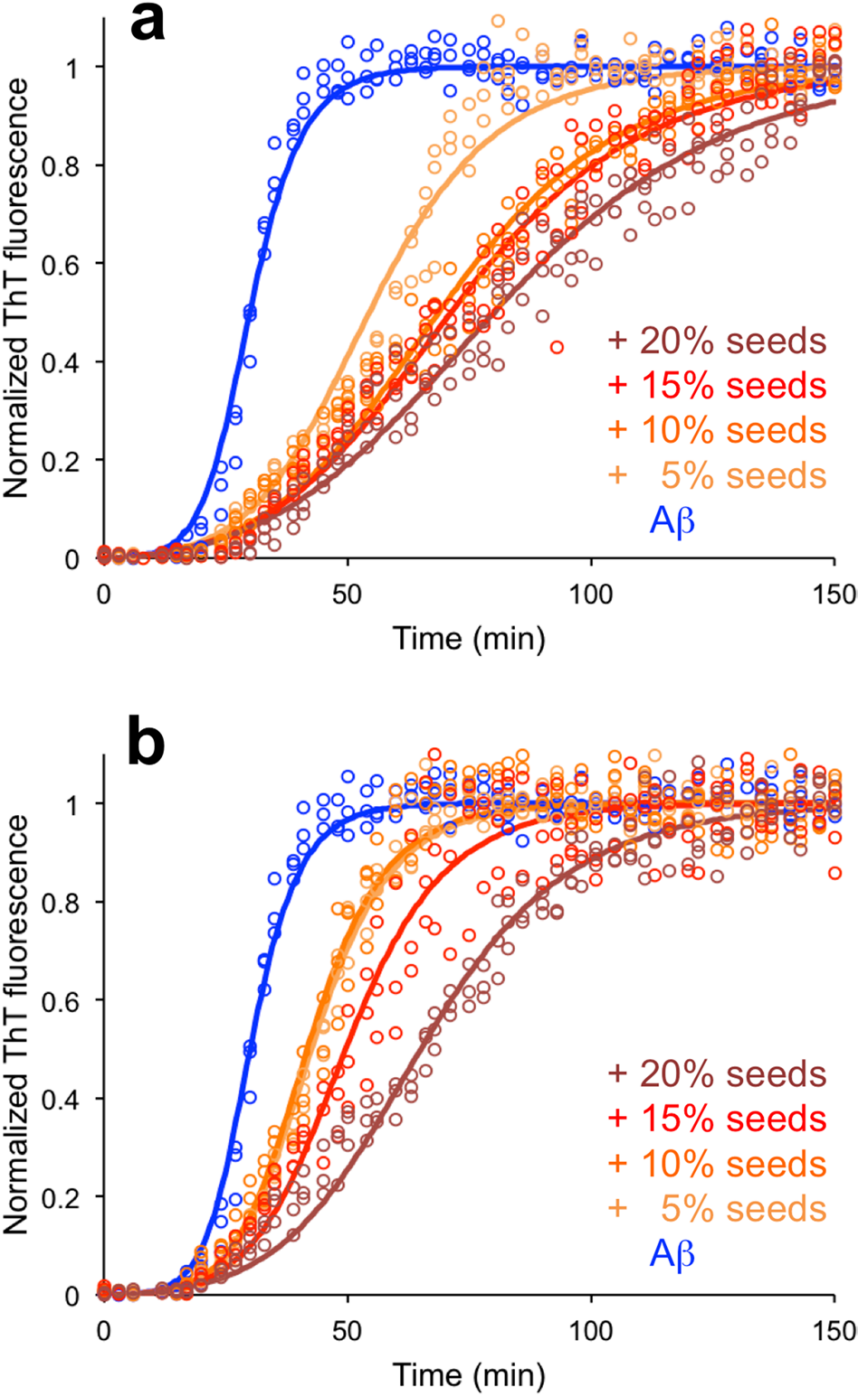
AmyloFit analysis of Aβ_1–42_ seeded with 5%, 10%, 15% and 20% seeds of (**a**) lysozyme and (**b**) oat protein. Experimental data for three replicates at each condition (circles) and the model fit (solid lines) are shown.

Two of the tested seed preparations, lysozyme and oat, appeared to have slightly stronger effects on the kinetics of Aβ_1–42_ aggregation. In both cases the addition of seeds delayed the assembly process. Inhibition of disease-associated amyloid formation by food protein fibrils has previously been reported for fish parvalbumin^48^. To acquire more insights in these effects we examined the ThT kinetics with two additional concentrations of seeds (15% and 20%). In both cases, a concentration-dependence was observed over the whole range of seed concentrations (Fig. 4). The shapes of the kinetic traces change in a way that more or less preserve the lag time but decrease the slope of the transition (i.e. makes it less sharp). The data was further analyzed using the AmyloFit modeling framework^35^. As mentioned above, the fibrillation kinetics for Aβ_1–42_ (without any seeds) is consistent with a secondary nucleation dominated model. This model is described by three rate constants: *k*_*n*_, *k*_+_ and *k*_*2*_, relating to primary nucleation, elongation and secondary nucleation, respectively. In a data set with unseeded reactions, the rate constants are not independent and appear as the products *k*_+_*k*_*n*_ and *k*_+_*k*_*2*_. The kinetics could also be affected if the added component sequester Aβ peptide monomers and thereby reduce the effective monomer concentration (*m*_*0*_). Attempts to reproduce the delayed fibrillation by letting one parameter (*m*_*0*_ or one of the rate constant products) vary while keeping the other parameters constant to the best fit values of Aβ_1–42_ alone were not successful. The best results were obtained for variation of the *k*_+_*k*_*2*_ parameter indicating that the inhibitory effect of lysozyme and oat seeds may be related to the elongation or secondary nucleation processes. Moreover, good fits could be obtained with variations in the *k*_+_*k*_*2*_ parameter if a second parameter (e.g. the size of the primary nucleus) were simultaneously refitted globally (Fig. 4) This was not the case for *m*_*0*_ or *k*_+_*k*_*n*_. Taken together, these results indicate that the inhibition mechanism is not specifically targeting one step in the assembly mechanism, as for instances observed for the BRICHOS chaperone^49^. The fact that the changes are better described by variation in *k*_+_*k*_*2*_ than in *k*_+_*k*_*n*_ or *m*_*0*_ suggests that the inhibition mechanism involves interactions between the seeds and Aβ *aggregates*, rather than the monomer.

To test if the inhibition mechanisms relied on electrostatic interactions we performed additional experiments in which the ionic strength of the samples was varied (see the Supporting information for details). We found that the aggregation kinetics of Aβ_1–42_ in the presence of 10% of lysozyme or oat seeds is strongly dependent on the salt concentration of the samples (Supporting information Fig. S1). This is, however, also the case for Aβ_1–42_ without any added seeds (Supporting information Fig. S1), which makes it difficult to draw any definitive conclusions regarding the role of electrostatic interactions. Nevertheless, we do observe a decreasing trend for the difference in aggregation half-time (*t*_*1/2*_ with seeds minus *t*_*1/2*_ without seeds) when the salt concentration is increased, which may be due to weaker electrostatic forces at high ionic strength (Supporting information Fig. S2).

### Seeding by fibrils prepared from food protein peptides

The general ability of proteins to form amyloid-like fibrils when exposed to high temperature under acidic conditions seems to be related to the promotion of protein hydrolysis under such conditions^32,50^. Hydrolysis is normally observed in parallel with fibril assembly and the formed fibrils have been shown to consist of shorter fragments of the originating proteins^30–32,51^. The fibrillation-promoting effects of hydrolysis can be understood in terms of more favorable thermodynamics of assembly for shorter polypeptide chains^8,52^. The typical pH for in vitro assembly is indeed similar to the conditions during food digestion in the stomach. Although the temperature is much lower, hydrolysis of proteins is instead promoted by enzymatic activity. Little is known about the potential assembly of degraded food proteins into amyloid-like structures during digestion. However, peptides corresponding to parts of human amyloid proteins (IAPP or transthyretin) were shown to accelerate amyloid deposition in a mouse model of AA amyloidosis^53^.

Synthetic variants of peptides that have been identified as core building blocks for amyloid fibrils often form fibrils on their own. In those cases, much milder conditions can be used than for the full-length proteins, which support the hypothesis that high temperature is primarily needed to unfold and hydrolyze the proteins. We prepared amyloid-like fibrils from two peptides corresponding to the core region of β-lactoglobulin amyloid (same region but different lengths) and from four different peptides corresponding to amyloid fibrils derived from soybean protein isolate.

**Figure 5:**
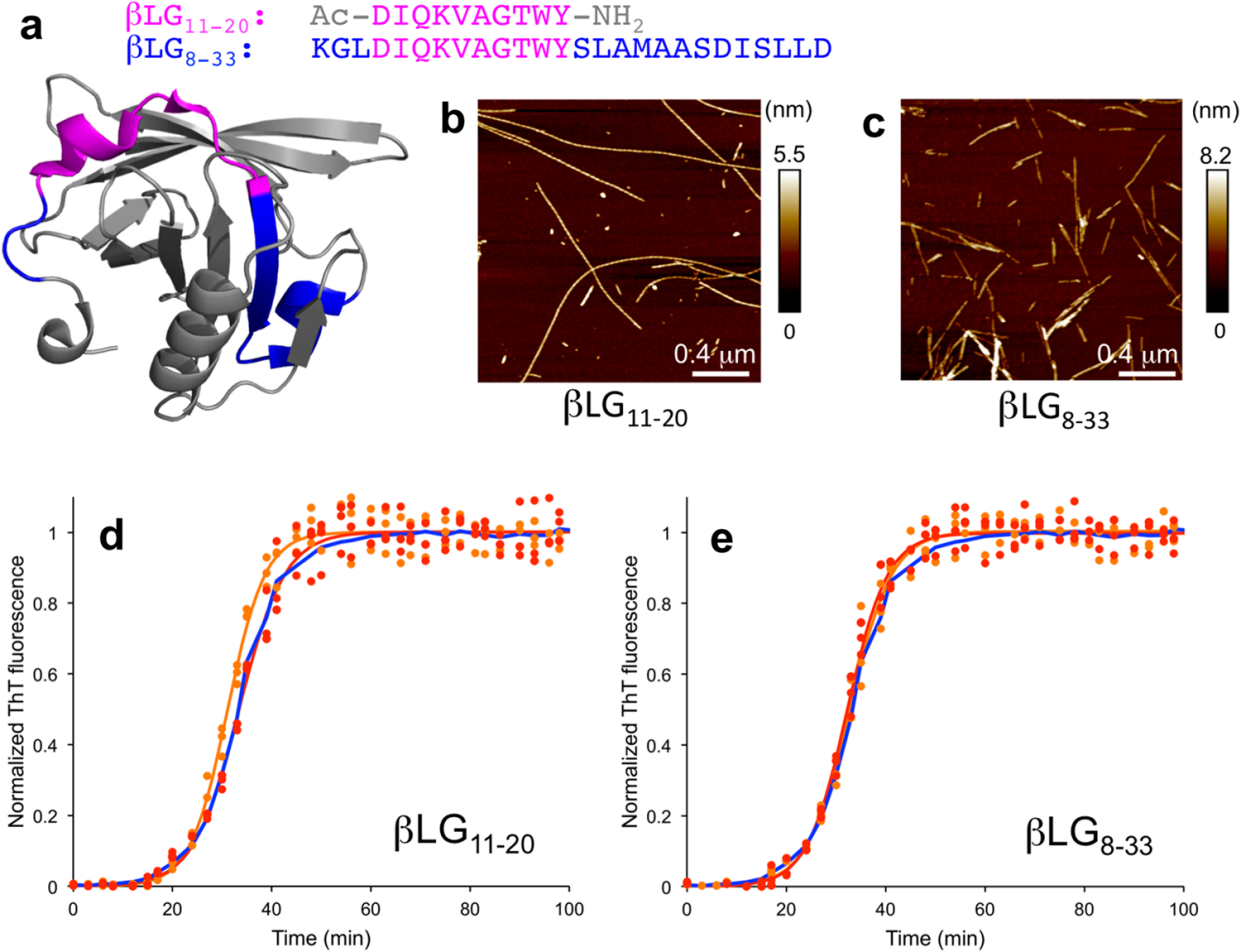
Seeding with amyloid fibrils from β-lactoglobulin-derived peptides. (**a**) Sequences and locations of the peptides in the native structure of β-lactoglobulin. (**b**,**c**) AFM images of amyloid fibrils from the (**b**) βLG_11-20_ and (**c**) βLG_8-33_ peptides, respectively. (**d**,**e**) ThT kinetic traces for Aβ_1–42_ without seeds (blue) and seeded with 5% (orange) or 10% (red) seeds from (**d**) βLG_11-20_ and (**e**) βLG_8-33_, respectively. Experimental data for three replicates at each condition (filled circles) and a sigmoidal curve fit of the average data (solid lines) are shown.

The *βLG*_*11-20*_* peptide* (residues 11–20 from β-lactoglobulin, Fig. 5a) has been shown to form amyloid-like fibrils at pH 7 with 5 M urea^54^ and at pH 2 (Fig. 5b)^28^. It is part of the longer *βLG*_*8-33*_* peptide* (residues 8–33, Fig. 5a) that has been identified as a core component of β-lactoglobulin fibrils formed at pH 2^32,51^. The βLG_8-33_ peptide forms amyloid-like fibrils when incubated at pH 2 (Fig. 5c). The seeds prepared from the βLG_11-20_ or βLG_8-33_ fibrils did not significantly affect the kinetics of Aβ_1–42_ fibrillation (Fig. 5d,e).

**Figure 6:**
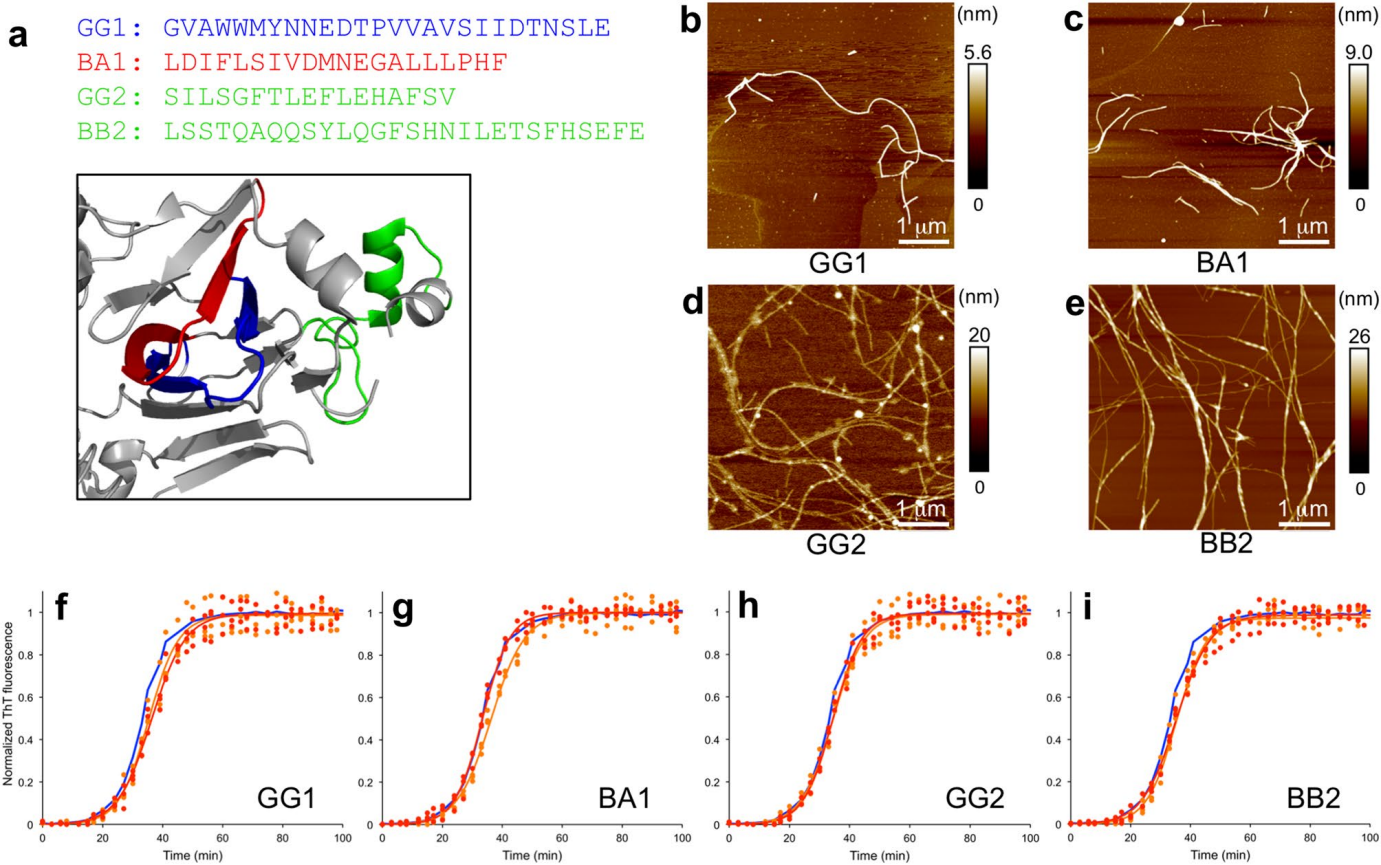
Seeding with amyloid fibrils from soy protein-derived peptides. (**a**) Sequences and location in the native structure of soy storage proteins. GG1, and GG2 originates from glycinin, BA1 from β-conglycinin subunit α, and BB2 from β-conglycinin subunit β. (**b**–**e**) AFM images of amyloid fibrils from the (**b**) GG1, (**c**) BA1, (**d**) GG2 and (**e**) BB2 peptides, respectively. (**f**–**i**) ThT kinetic traces for Aβ_1–42_ without seeds (blue) and seeded with 5% (orange) or 10% (red) seeds from (**f**) GG1, (**g**) BA1, (**h**) GG2 and (**i**) BB2 peptides, respectively. Experimental data for three replicates at each condition (filled circles) and a sigmoidal curve fit of the average data (solid lines) are shown.

In our previous study of amyloid-fibrils from soy protein we identified five core peptides of these fibrils^30^. The peptides originate from different protein subunits in the isolate (glycinin or β-conglycinin) and from different parts of the native protein structure (Fig. 6a). The GG1 and BA1 peptides correspond to “β-arches” in the native state while GG2 and BB2 originates for similar helical segments but in different protein subunits. For four of these five peptides it was possible to produce amyloid-like fibrils from synthetic peptide starting material (Fig. 6b-e) and all four peptide amyloids were here investigated in the Aβ aggregation kinetics assay. However, none of the peptide fibril seeds were able to change the Aβ_1–42_ fibrillation kinetics in the ThT assays (Fig. 6).

## Conclusions

**Figure 7:**
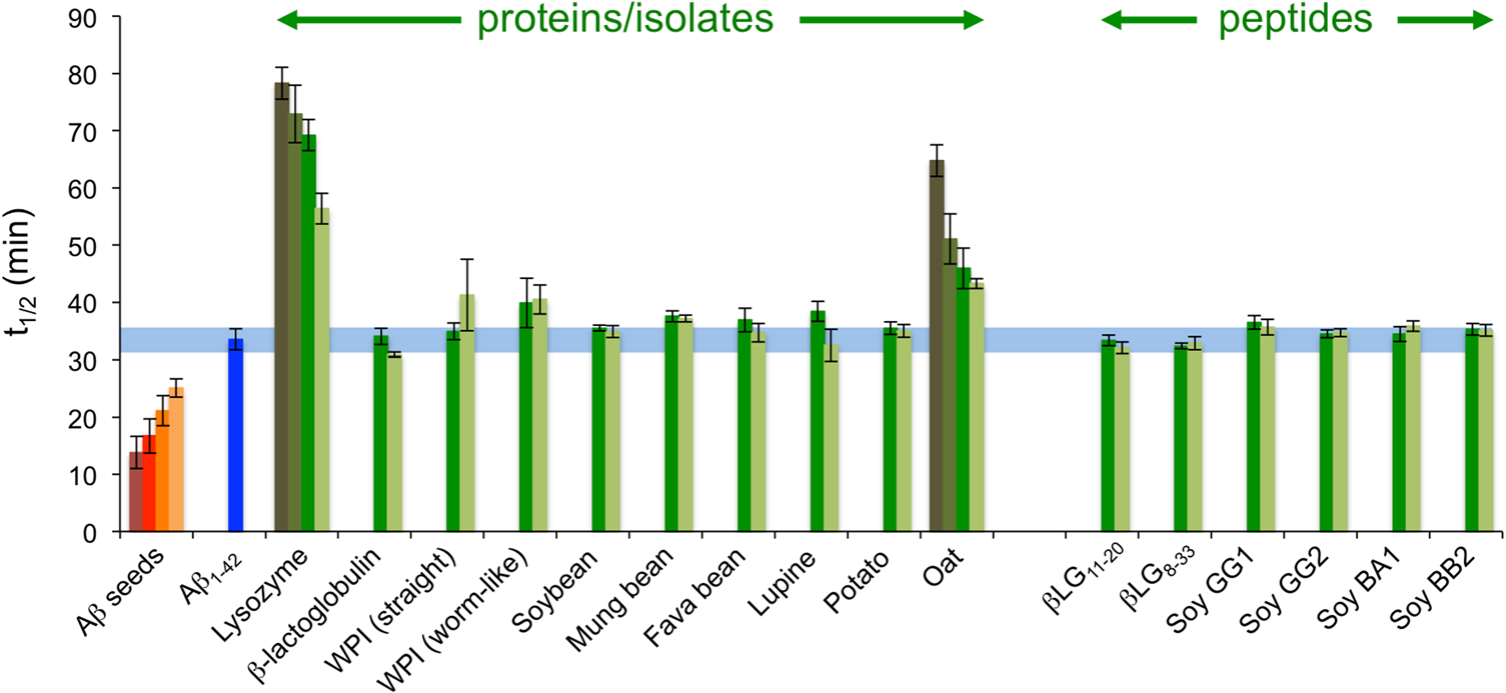
Summary of *t*_*1/2*_ for all seeding conditions presented in this study. For each type of seed the values are shown from the highest concentration (left) to the lowest concentration (right). The blue horizontal region corresponds to *t*_*1/2*_ for unseeded Aβ_1–42_ ± standard deviation.

In this study we have investigated the effects of 16 different food protein amyloid seeds on the kinetics of Aβ_1–42_ amyloid formation. The seeds were obtained from animal proteins, plant proteins and synthetic peptides corresponding to aggregation prone segments of animal or plant proteins. While it was demonstrated that Aβ-seeds triggered a faster assembly process, acceleration of Aβ_1–42_ aggregation was not observed in any of the experiments with added food protein amyloid seeds (Fig. 7). In at least two cases, clear inhibition of the fibrillation was observed. The mechanism for the inhibitory effect seems to be related to interactions between Aβ *fibrils* and the food protein amyloid seeds, possibly mediated by electrostatic forces. The results support the view that amyloid structures from food-derived proteins do not constitute a health risk although more studies on different disease-associated proteins are still needed. It should also be noted that there may be a significant difference between amyloid formed in vitro and amyloid originating from in vivo deposits, as illustrated by the enhanced aggregation of Aβ triggered by AA from cattle^24^. The amyloid-like structures applied in materials, or even food, however, falls within the in vitro category.

## Materials and methods

### Protein/peptide sources

Hen egg white lysozyme was purchased from Merck (cat.# 105281) and bovine β-lactoglobulin from Sigma (cat.# L0130). Whey protein isolate (Lacprodan DI-9224) was obtained from Arla Food Ingredients, soy protein isolate (SUPRO 120 IP) from Solae Belgium N.V., and potato protein isolate (85043, Batch 1502000052) from Lyckeby Starch AB (Kristianstad, Sweden). Fava bean (*Vicia faba* minor; cv. Gloria) cultivated in Sweden was provided by RISE (Research Institutes of Sweden). Mung bean (*Vigna radiate*) cultivated in Myanmar was provided by Lantmännen (Sweden). Lupine seeds (*Lupineus angustifolius*, cv. Boregine) were bought from Italy. Milled, defatted, and air-classified oat (*Avena sativa* cv. Mathilda) flour was provided by Lantmännen (Sweden). Protein isolates were prepared as described in our previous work^13^. The synthetic peptides derived from soy proteins were purchased from Alexotech AB (Umeå, Sweden).

### Production of Aβ_1–42_

Aβ_1–42_ was produced as a fusion protein with N-terminal domain (NT) of spider silk protein and purified as described previously^33^. Briefly, NT*_FISp_-Aβ_42_ fusion protein with a TEV recognition site in-between was expressed in *E. coli* BL21 (DE3) and purified in 8 M urea. Ni^2+^ affinity chromatography using 2 × 5 mL Ni–NTA column (Cytiva) was performed to capture the fusion protein. His6-NT*_FISp_ was cleaved from Aβ_1–42_ by cleaving the TEV site with TEV protease. Aβ_1–42_ was separated from the fusion partner on a Superdex 30 26/600PG size exclusion column (Cytiva). The sample was aliquoted into 1 mL of 80 µM fractions, lyophilized, and stored at − 80 °C.

### Production of peptides derived from β-lactoglobulin

The βLG_11-20_ and βLG_8-33_ peptides were synthesized on a single channel Biotage Alstra Initiator in a 10 mL reaction vessel. All F-moc-protected amino acids, reagents, and solvents were of analytical grade and purchased from Sigma-Aldrich/Merck unless otherwise indicated. The chemicals were used as received without any further purification. Rink amide ChemMatrix resin (0.2 g) with a loading capacity of 0.50 mmol/g was placed in the reaction vessel and swollen in 4.5 mL of *N,N*-dimethylformamide (DMF) for 20 min with micro-wave heating at 70 °C. Appropriate amounts of each F-moc-protected amino acid were dissolved in *N*-methylpyrrolidone (NMP) to a final concentration of 0.5 M. The coupling reactions were carried out using 5 equivalents of N,Nʹ-Diisopropylcarbodiimide/Oxyma (0.5 M) in DMF. Each coupling reaction was carried out for 7.5 min with micro-wave heating at 70 °C. All coupling reagents and amino acids were used in excess (5 eq.) to get sufficient coupling. After deprotection with 20% piperidine in NMP for 3 min, the resin was washed four times with DMF. The completion of coupling and deprotection reactions were verified by the Kaiser Test (ninhydrin) at intervals. The peptide was cleaved from the resin using 95% trifluoroacetic acid (TFA), 2.5% triisopropyl silane (TIS), and 2.5% water and isolated by precipitation in cold diethyl ether. The mass of the product was verified by MALDI-TOF analysis. The crude peptide was then purified by reverse-phased HPLC, containing semi-preparative Sepax Poly-RP column (5 µm, 300 Å, 4.6 × 250 mm). Mobile phases were 0.1% TFA in H_2_O (A) and 0.1% TFA in acetonitrile (B). The purification was carried out at 50 °C with a flow rate of 3.0 mL/min and a gradient of 20–60% B over 40 min.

### Aβ_1–42_ fibrillation

Lyophilized Aβ_1-42_ peptide was dissolved to 40 µM in 20 mM HEPES, 140 mM NaCl, pH 8 into a 1.5 mL LoBind tube (Eppendorf). Fibrils were allowed to form at 37 °C overnight without agitation. The presence of fibrils was confirmed by ThT binding assay and AFM.

### Fibrillation of food proteins and peptides

#### Lysozyme fibrillation

Lysozyme powder was dissolved in 50 mM NaH_2_PO_4_, pH 2 to a final concentration of 5.6 mg/mL. To pellet any existing aggregates, the solution was centrifuged at 3900 × g for 10 min. The supernatant was transferred to a new 1.5 mL LoBind tube (Eppendorf). The tube was incubated at 80 °C at 700 rpm in a Thermomixer (Eppendorf) for 25 h to allow for fibril formation^40^. The presence of fibrils was confirmed by ThT fluorescence and AFM.

#### β-lactoglobulin fibrillation

β-lactoglobulin powder was dissolved in 10 mM HCl pH 2 to a final concentration of 20 mg/ml. The solution was centrifuged 18,500×*g* for 30 min and filtered through a polyethersulfone (PES) filter with 0.45 um pore size. The sample was then incubated at 80 °C for 24 h to produce fibrils. The presence of fibrils was confirmed by ThT fluorescence and AFM.

#### Fibrillation of food protein isolates

Fibril formation from the protein isolates (commercial or prepared in-house) was carried out at pH 2 and 85–90 °C as described in earlier work (see references in Table 1). The presence of fibrils was confirmed by ThT fluorescence and AFM.

#### Fibrillation of β-lactoglobulin-derived peptides

Fibrillation of βLG_11-20_ peptide was achieved as described in Ref.^28^. Briefly, the peptide was dissolved (1 mg/ml) in 10 mM HCl containing 5% β-LG seeds. The solution was centrifuged at 15,600×*g* and the supernatant solution was incubated at 50 °C and 500 rpm agitation for 48 h in a Thermomixer (Eppendorf) and then at 50 °C without agitation for two more days. The fibrils were purified using spin filtration with 10 kDa cut-off (GE Healthcare). The presence of fibrils was confirmed by ThT fluorescence and AFM.

The βLG_8-33_ peptide was dissolved to 1 mg/mL in 10 mM HCl and centrifuged at 15,600×*g*. The supernatant solution was incubated at 50 °C for 48 h at an agitation of 300 rpm in a Thermomixer (Eppendorf). The solution was then further incubated for 48 h without agitation. The fibrils were purified by spin filtering with 100 kDa cut-off (GE Healthcare). The presence of fibrils was confirmed by ThT fluorescence and AFM.

#### Fibrillation of soy protein-derived peptides

Saturated solutions for each soy peptide were prepared in 10 mM HCl (pH 2) to a final volume of 1.5 mL. To remove insolubilized material, all the aliquots were centrifuged at 13,500×*g* for 45 min. Each of the peptide solutions was incubated at 50 °C for 5 days in quiescent conditions using a Thermomixer (Eppendorf). The fibrils were purified using spin filtration with 10 kDa cut-off (GE Healthcare). The presence of fibrils was confirmed by ThT fluorescence and AFM.

### Atomic force microscopy of amyloid fibril

AFM was carried out using a Dimension FastScan AFM instrument (Bruker) operating in tapping mode and using FastScan A probes (Bruker). Fibril morphology was investigated in tapping mode, using samples that were diluted between 1:50 and 1:1000 in 10 mM HCl. After dilution, 25 μL of each sample was deposited onto freshly cleaved mica and incubated for 30 min at room temperature. After incubation, samples were washed using distilled water and dried using a stream of compressed air. Alternatively, 25 μL of the sample was applied on the mica surface and left to dry at ambient conditions. Nanoscope analysis software (Version 1.5 or 1.9, Bruker) was used to process the image data by flattening the height topology data and removing the tilt and scanner bow.

### Preparation of amyloid seeds

Food amyloid fibrils were diluted to 0.1 mg/mL in 10 mM HCl, pH 2 in 1.5 mL LoBind tube (Eppendorf). To produce seeds, the sample was sonicated using a Qsonica Q500 sonicator equipped with a 6 mm micro tip for a total of 30 s (20% amplitude, 2 s on and 10 s off). The resulting seed solution was immediately transferred onto ice. Aβ_1–42_ seeds were produced in the same procedure but the samples were diluted in 20 mM HEPES, 140 mM NaCl, pH 8.

### Cross-seeding aggregation kinetics assays

To monitor the cross-seeding aggregation kinetics, a 320 µL samples containing 3 µM (0.014 mg/mL) Aβ_1–42_ peptide, 0%, 5% or 10% (w/w) of amyloid seeds (from a 0.1 mg/mL stock), and 15 µM ThT (from a 1 mM stock) were prepared in 20 mM HEPES, 140 mM NaCl, pH 8. The samples were prepared on ice and quickly dispensed in triplicates into a 96-well half-area clear bottom and nonbinding polystyrene plate (CORNING; cat# 3881) at a final volume of 100 μL in each well and incubated at 37 °C under quiescent condition. Fibrillation was monitored by measuring ThT fluorescence through the bottom of the plate at every 180 s with a 440 nm excitation filter and a 480 nm emission filter using a CLARIOstar (BMG Labtech, Offenburg, Germany). For Aβ self-seeding, the same procedure was used. The kinetics data was normalized after linear corrections of the pre- and post transition baselines.

## Supplementary Information

Supplementary Information.

## Data Availability

The datasets generated during and/or analyzed during the current study are available from the corresponding author on reasonable request.
