# Supplementary material for: Food protein-derived amyloids do not accelerate amyloid β aggregation
Source: Sci Rep. 2023 Jan 31;13:985. doi: 10.1038/s41598-023-28147-5 (PMC9889329; doi:10.1038/s41598-023-28147-5)
Supplement: Supplementary file 1 — Supplementary Information. [file 41598_2023_28147_MOESM1_ESM.pdf]

SUPPORTING INFORMATION

Food protein-derived amyloids do not accelerate amyloid β aggregation

*M. Mahafuzur Rahman[1], Rodrigo Sanches Pires[1], Anja Herneke[2], Vasantha Gowda[1], Maud Langton[2], Henrik Biverstål[3], Christofer Lendel[1]**

[1] Department of Chemistry, KTH Royal Institute of Technology, Teknikringen 30, SE-100 44, Stockholm, Sweden.

[2] Department of Molecular Sciences, Swedish University of Agricultural Sciences, BioCentrum, Almas allé 5, SE-756 61, Uppsala, Sweden.

[3] Department of Biosciences and Nutrition, Karolinska Institutet, NEO/Floor 8, Blickgången 16, SE-141 52 Huddinge, Sweden.

Supporting methods

Cross-seeding aggregation kinetics assays at different ionic strengths

To investigate the role of electrostatic interactions in the inhibition mechanisms of lysozyme and oat amyloid seeds, $A\beta_{1\text{-}42}$ aggregation kinetics was monitored in a series of samples with different concentrations of NaCl (5 mM, 50 mM, 100 mM, 140 mM and 200 mM). The experiments were performed with 10% lysozyme or oat seeds. In addition, control samples with only $A\beta_{1\text{-}42}$ peptide and the same NaCl concentrations were included. All samples were investigated in triplicates. The seed preparation and the experimental procedures were the same as described in the main text.

Supporting figures

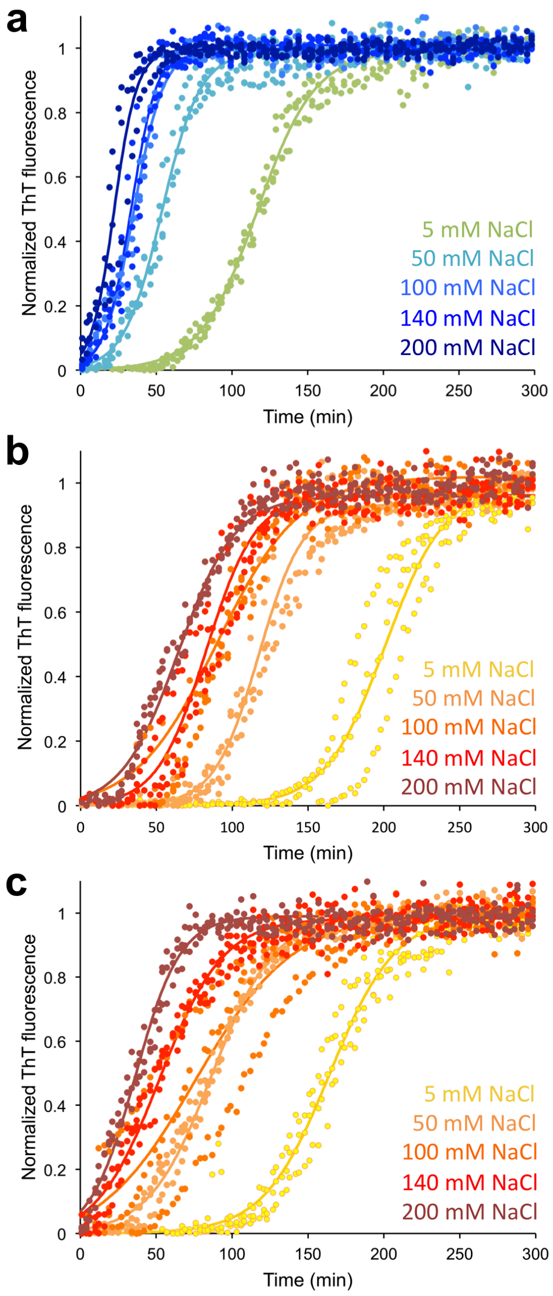

Figure 1. ThT aggregation kinetics of $A\beta_{1\text{-}42}$ at different ionic strengths. (**a**) $A\beta_{1\text{-}42}$ without any added seeds. (**b**) $A\beta_{1\text{-}42}$ with 10% lysozyme seeds. (**c**) $A\beta_{1\text{-}42}$ with 10% oat protein seeds. For each sample condition, experimental data for three replicates at each condition (filled circles) and sigmoidal curve fits of the average data (solid lines) are shown.

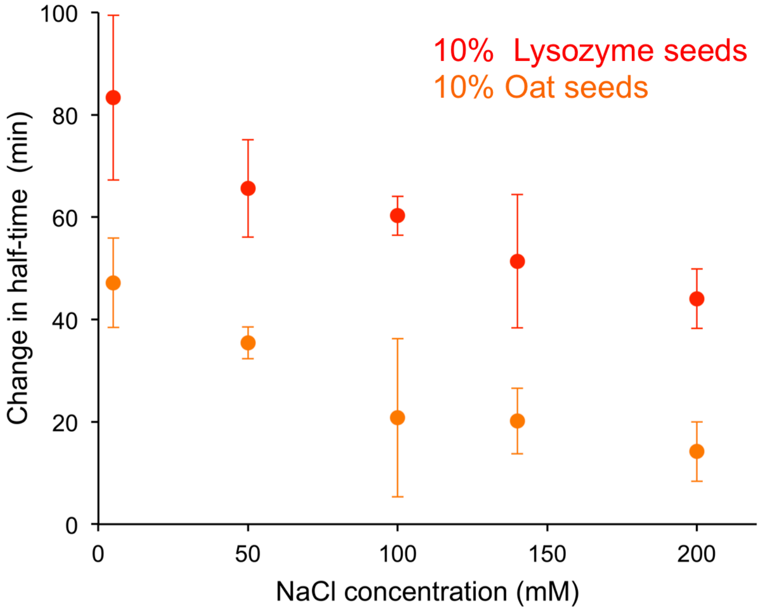

Figure S2. Change in aggregation half times ($t_{1/2}$), i.e. the difference between $t_{1/2}$ with 10% seeds added and $t_{1/2}$ for $A\beta_{1\text{-}42}$ without any seeds, as function of the salt concentration in the sample. Average values ± standard deviations are shown.
